# 27-gauge and 25-gauge vitrectomy day surgery for idiopathic epiretinal membrane

**DOI:** 10.1186/s12886-017-0585-1

**Published:** 2017-10-10

**Authors:** Saigen Naruse, Hiroyuki Shimada, Ryusaburo Mori

**Affiliations:** 1 Miyahara Ophthalmological Clinic, Saitama City, Saitama Japan; 2 Department of Ophthalmology, Nihon University Hospital, 1-6 Surugadai, Kanda, Chiyodaku, Tokyo, 101-8309 Japan

**Keywords:** Angled incision, Day surgery, Central retinal thickness, Hypotony, Idiopathic epiretinal membrane, Ocular hypertension, Operation time, Postoperative complication, Straight incision, 25-gauge vitrectomy, 27-gauge vitrectomy, Visual acuity

## Abstract

**Background:**

This study compared the postoperative outcomes of 27-gauge (G) and 25-G vitrectomy performed for the treatment of idiopathic epiretinal membrane (ERM).

**Methods:**

The study design was single center, retrospective, interventional case series. Two hundred consecutive eyes that underwent primary vitrectomy for ERM (27-G vitrectomy in 100 eyes and 25-G vitrectomy in 100 eyes) were studied for 6 months. In all eyes, scleral tunnels were made using angle incisions, and air or gas exchange was performed.

**Results:**

There were no significant differences in age, spherical diopter power, as well as preoperative Early Treatment Diabetic Retinopathy Study (ETDRS) score, central retinal thickness (CRT), and intraocular pressure between the 27-G and 25-G groups. The proportions of simultaneous cataract surgery (27-G vs. 25-G: 82% vs. 90%), air-filled eyes (99% vs. 98%), and scleral wound suture at the end of surgery (0% vs. 0%) were not significantly different between two groups. The mean operation time for vitrectomy was significantly (*P* = 0.0322) longer by 4 min for 27-G (37 min) compared to 25-G (33 min) vitrectomy. Gain in ETDRS score was significantly (*P* = 0.0421) better in 27-G group (4.7 ± 8.1 letters) compared to 25-G group (1.1 ± 13.6 letters) at 1 month post-vitrectomy, but not significantly different at 3 and 6 months (*P* = 0.0835 and 0.0569, respectively). Decrease in CRT was significantly (*P* = 0.0354) greater in 27-G group (−24.2 ± 50.0 μm) compared to 25-G group (−8.0 ± 48.6 μm) at 1 month post-vitrectomy, but not significantly different at 3 and 6 months (*P* = 0.6059 and 0.1725, respectively). On postoperative day 1, hypotony (≤ 6 mmHg) was observed in 2 eyes in 27-G group and 6 eyes in 25-G group, while ocular hypertension (≥ 25 mmHg) was found in 4 eyes in 27-G group and 11 eyes in 25-G group, with no significant differences between two groups. Postoperative complications requiring treatment occurred in one eye (vitreous hemorrhage) in 27-G group, and in two eyes (vitreous hemorrhage and retinal detachment in one eye each) in 25-G group.

**Conclusions:**

Although 27-G vitrectomy requires operation time of 4 min longer compared to 25-G vitrectomy for ERM surgery, using the 27-G system results in earlier recovery of visual acuity, CRT improvement and stabilized ocular pressure.

## Background

Transconjunctival microincision vitrectomy surgery (MIVS) using 23-gauge (G), 25-G or 27-G instrumentation has gained popularity rapidly since the development of wide-angle viewing systems [1–3]. In 2010, Oshima and associates described the initial feasibility and safety of a novel 27-G MIVS system [3]. Even for 27-G vitrectomy, the indications have been expanded to include proliferative diabetic retinopathy, retinal detachment, and proliferative vitreoretinopathy [4, 5]. However, the number of studies on 27-G vitrectomy remains small [6].

Studies using 20-G, 23-G and 25-G vitrectomy have reported that using smaller gauge instrument results in few postoperative inflammation [7], less surgically induced astigmatism [8, 9], stable postoperative intraocular pressure (IOP) [10, 11], and more rapid recovery of visual acuity after operation [10–13]. Using angled incision instead of straight incision to make sclerotomy, and using air or gas exchange instead of fluid exchange at the end of operation have been reported to be effective in stabilizing postoperative IOP [14–22]. In the present study, we performed a retrospective study on 200 eyes undergoing vitrectomy for idiopathic epiretinal membrane to compare the postoperative outcomes of 27-G and 25-G surgery using angled incision and air exchange performed as day surgery.

## Methods

### Patients

In this retrospective study, 200 eyes of 200 patients (91 females, 109 males) that underwent primary vitrectomy day surgery for idiopathic epiretinal membrane between January 2012 and August 2016 were studied. The study adhered to the tenets of the Declaration of Helsinki. This study was approved and consented to participate’ section any permissions obtained to use/analyse the patients’ records by the Ethical Committee of the Nihon University School of Medicine (Number 20161203). The mean patient age was 68.5 ± 9.3 (range, 44 to 96) years. Informed consent was obtained from each subject following an explanation of the vitrectomy procedures and potential adverse effects of the procedure. All surgeries were performed by two surgeons (H.S. and S.N.). H.S. performed 56% of the 25-G and 51% of the 27-G vitrectomies. The proportions of surgeries performed by each surgeon did not differ between 25-G and 27-G vitrectomies (*P* = 0.5707). There were no significant differences in operation time, surgical indications, surgical methods, and surgical outcome between the series performed by the two surgeons. Patients requiring general anesthesia and systemic management, and patients who desired inpatient treatment were excluded from the study.

### Vitrectomy

Vitrectomy was conducted using the Constellation® Vison System (Alcon Laboratories, Fort Worth, TX). Preoperative antisepsis by ocular instillation of topical antibiotics (levofloxacin: Shionogi, Osaka, Japan) six times a day was started the day before surgery. One hundred consecutive eyes were operated with 25-G vitrectomy (Alcon Surgical) between January 2012 and December 2014 (25-G group). Another 100 eyes were operated with 27-G vitrectomy (Alcon Surgical), between January 2015 and August 2016 (27-G group).

Vitrectomy was performed under retrobulbar anesthesia in all patients. An antibiotic (flomoxef sodium, Shionogi, Osaka, Japan) was infused intravenously during surgery. After placing the lid speculum, the operative field was irrigated with 0.25% povidone-iodine [23]. The 0.25% povidone-iodine solution was freshly prepared before surgery, by diluting 10% povidone-iodine with sterile physiological saline. Using forceps, the conjunctiva was displaced slightly toward the cornea [24]. Incisions were made to insert three valved cannula trocar systems obliquely at an angle of 30° and parallel to the limbus in a 1-step procedure [14]. 25-gauge vitrectomy was performed in all cases using a cut rate of 5000 cuts per min (cpm) and linear aspiration of 0–650 mmHg. 27-gauge vitrectomy was performed in all cases using a cut rate of 7500 cpm and linear aspiration of 0–650 mmHg. For posterior visualization, RESIGHT 700 (Carl Zeiss Meditec AG, Oberkochen, Germany) was used. During vitrectomy, the operative field was flushed repeatedly with infusion fluid or 0.25% povidone-iodine.

Epiretinal membrane and macular hole were treated using 25-G or 27-G internal limiting membrane forceps (Alcon Laboratories) and a plano-concave contact lens (Hoya, Tokyo, Japan). No chandelier light source and no scleral buckling were used in all patients. Peripheral vitreous was excised until the cannula tip was exposed [25]. At the completion of surgery, 30% of the vitreous volume was replaced with air, and care was taken to ensure air tightness to facilitate early closure of the scleral wound. After removing each cannula, the sclerotomy roof was compressed on both sides with the forceps tip to close the scleral wound [14], and air pressure was increased to 30 mmHg to close the sclerotomy floor. At completion of surgery, a triangular surgical spear was used to check for vitreous prolapse at all three ports. When prolapse of transparent vitreous through the scleral wound was detected, the prolapsed vitreous was excised with a cutter. Gas tamponade, when used, was performed using17% sulfur hexafluoride (SF6; Alcon Laboratories) or 9% perfluoropropane (C3F8; Alcon Laboratories). Finally, the operative field was irrigated with 0.25% povidone-iodine, and subconjunctival steroid (dexamethasone; Wako, Tokyo, Japan) and antibiotic (tobramycin; Shionogi, Osaka, Japan) were injected. Operation time was defined as the time taken to perform vitrectomy. When simultaneous cataract was performed, the time taken for vitrectomy only was measured.

Simultaneous cataract surgery was conducted in patients 50 years of age or older because cataract tends to progress after vitrectomy. Cataract surgery was conducted using two types of viscoelastic materials; Viscoat (Alcon Laboratories) and Healon (AMO, Uppsala, Sweden). Phacoemulsification (Constellation; Alcon Laboratories) was performed through an incision in the superior cornea. A foldable intraocular lens (SN60WF; Alcon Laboratories) was inserted inside the capsule. Cataract surgery was conducted through a superior corneal incision. Scleral and corneal wound was closed with one nylon 10-0 suture, which was removed 1 week later.

### Pre and postoperative examinations

Patients were examined before, 1 to 2 days, 1 week, 2 weeks, 1 month, 2 months, 3 months and 6 months after surgery. Hypotony was defined as an IOP of 6 mmHg or lower [5], and ocular hypertension was defined as an IOP of 25 mmHg or higher [5]. Corneal epithelial damage, anterior chamber inflammation, vitreous inflammation and fundus examination were assessed at each postoperative follow-up using a slit lamp microscope and indirect ophthalmoscopy. Postoperative complications including hypotony, ocular hypertension, retinal detachment, endophthalmitis, and choroidal detachment were also detailed if present. Visual acuity was measured using the Landolt ring chart, and the result was converted to Early Treatment Diabetic Retinopathy Study (ETDRS) score for analysis. Gain of ETDRS score after surgery (postoperative ETDRS score – preoperative EDTRS score) was also analyzed. Central retinal thickness (CRT) was measured using optical coherence tomography (OCT). Decrease in CRT after surgery [postoperative CRT (μm) – preoperative CRT μm)] was also analyzed.

### Outcome measures

The outcome measures were intraoperative complications, wound closure at the end of surgery, operation time for vitrectomy, IOP on postoperative day 1 and day 7, complications occurring up to 6 months after surgery, as well as visual acuity and CRT at 1 month, 3 months and 6 months after surgery.

### Statistics

Statistical analyses were performed using SPSS software version 21 (SPSS, Inc., Chicago, IL). Values are expressed as mean ± standard deviation (SD) or percentage. Chi-squared test for independent variable or Mann-Whitney test was used to compare two groups. *P* values less than 0.05 were considered to be statistically significant.

## Results

### Baseline data

The 27-G group and 25-G group did not differ significantly in baseline and operative characteristics (Table 1) including female/male ratio (27-G vs. 25-G: 49/51 vs. 42/58; *P* = 0.3200), age (67.6 ± 9.6 vs. 69.4 ± 8.9 years; *P* = 0.2498), pseudo-phakic/phakic ratio (14/86 vs. 10/90; *P* = 0.3841), preopertive ETDRS score (74.7 ± 12.5 vs. 73.6 ± 13 letters; *P* = 0.7298), preoperative CRT (381.5 ± 84.0 vs. 373.5 ± 97.3 μm; *P* = 0.3850), preoperative IOP (14.5 ± 2.8 vs. 14.5 ± 3.2 mmHg; *P* = 0.8422), and spherical diopter power (−1.5 ± 3.3 vs. -1.3 ± 3.6 D; *P* = 0.3314).

**Table 1:** Comparisons of demographic characteristics and baseline ocular features between 27- and 25-gauge vitrectomy

Surgical system used (n)	Female/male ratio	Mean age (range)	Pseudo-phakic/phakic ratio	Intraocular pressure	Spherical diopter power
27-gauge (100)	49/51	67.6 ± 9.6 yrs. (44–87)	14/86	14.5 ± 2.8 mmHg (8–25)	−1.5 ± 3.3 D
25-gauge (100)	42/58	69.4 ± 8.9 yrs. (49–96)	10/90	14.5 ± 3.2 mmHg (9–25)	−1.3 ± 3.6 D
*P* value	0.3200^a^	0.2498^b^	0.3841^a^	0.8422^b^	0.3314^b^

^a^Chi-squared test for independent variable, ^b^Mann-Whitney test

### Outcome measures

The 27-G and 25-G groups did not differ significantly in percent of simultaneous cataract surgery (82% vs. 90%; *P* = 0.1030) or percent of air-filled eye (99% vs. 98%; *P* = 0.5607) (Table 2). Operation time was significantly (*P* = 0.0323) longer by approximately 4 min in the 27-G group (36.7 ± 12.8 min) compared to the 25-G group (32.7 ± 10.1 min). There were no serious intraoperative complications in both groups. The scleral wound suture rate was 0% in both groups, with no difference. Postoperative complications consisted of retinal detachment (27-G vs. 25-G: 0% vs. 1%) and vitreous hemorrhage (1% vs. 1%), with no significant difference in incidence (*P* = 0.5607) between two groups. All three cases recovered by repeat vitrectomy.

**Table 2:** Comparisons of intraoperative parameters and postoperative outcomes between 27- and 25-gauge vitrectomy

Surgical system used (no. of eyes)	Surgical procedure (% of eyes)	Exchange procedure (% of eyes)	Operation time for vitrectomy (min) mean ± SD (range)	Wound suture at completion of surgery (% of eyes)	Postoperative complication (% of eyes)
27-gauge (100)	PEA + IOL + VIT (82%) VIT (18%)	Air (99%) 17% SF_6_ (1%)	36.7 ± 12.8 (15–88)	0/100 eyes (0%)	Vitreous hemorrhage (1%)
25-gauge (100)	PEA + IOL + VIT (90%) VIT (10%)	Air (98%) 17% SF_6_ (1%) 9%C_3_F_8_ (1%)	32.7 ± 10.1 (12–65)	0/100 eyes (0%)	Retinal detachment (1%) Vitreous hemorrhage (1%)
*P* value	0.1030^a^	0.5607^a^	0.0323^b^	>0.9999^a^	0.5607^a^

^a^Chi-squared test for independent variable, ^b^Mann-Whitney test

On postoperative day 1, the respective IOP for the 27-G and 25-G groups were 15.0 ± 5.8 and 16.3 ± 7.9 mmHg, hypotony rates were 2% and 6%, and ocular hypertension rates were 4% and 11%. Although IOP tended to be more stable in the 27-G group, there were no significant differences (Table 3). On postoperative day 7, the respective IOP for the 27-G and 25-G groups were 15.0 ± 5.1 and 15.5 ± 4.7 mmHg, hypotony rates were 0% and 0%, and ocular hypertension rates were 2% vs. 1%. Improvement in IOP compared to day 1 was observed in both groups, and the IOP in the 27-G group was apparently better although there was no significant difference.

**Table 3:** Comparisons of hypotony and ocular hypertension rates between 27- and 25-gauge vitrectomy

Surgical procedure	First postoperative day			Seven postoperative day		
	IOP (mmHg) mean ± SD (range)	Hypotony	Ocular hypertension	IOP (mmHg) mean ± SD (range)	Hypotony	Ocular hypertension
27-gauge vitrectomy (100 eyes)	15.0 ± 5.8 (5-41)	2/100 eyes (2%)	4/100 eyes (4%)	15.0 ± 5.1 (7-38)	0/100 eyes (0%)	2/100 eyes (2%)
25-gauge vitrectomy (100 eyes)	16.3 ± 7.9 (3-48)	6/100 eyes (6%)	11/100 eyes (11%)	15.5 ± 4.7 (6-41)	0/100 eyes (0%)	1/100 eyes (1%)
*P* value	0.2701^a^	0.1489^b^	0.0602^b^	0.2701^a^	>0.9999^b^	0.5607^b^

^a^Mann-Whitney test, ^b^chi-squared test for independent variable, *IOP* intraocular pressure. Hypotony was defined as an IOP of 6 mmHg or lower. Ocular hypertension was defined as an IOP of 25 mmHg or higher

The gain in ETDRS score was significantly better in the 27-G group compared to the 25-G group (27-G: 4.7 ± 8.1 letters vs. 25-G 1.1 ± 13.6 letters, *p* = 0.0421) at 1 month post-vitrectomy, and tended to be better at 3 and 6 months although the differences are not significant (*P* = 0.0835 and 0.0569, respectively) (Table 4). Decrease in CRT was significantly (*P* = 0.0354) greater in 27-G group (−24.2 ± 50.0 μm) compared to 25-G group (−8.0 ± 48.6 μm) at 1 month post-vitrectomy, but not significantly different at 3 and 6 months (*P* = 0.6059 and 0.1725, respectively). No postoperative endophthalmitis, sclerotomy-related retinal tears, and choroidal detachments were encountered in the 6-month follow-up period.

**Table 4:** Comparisons of pre- and post-operative visual acuity and central retinal thickness between 27- and 25-gauge vitrectomy

Surgical procedure	Preoperative ETDRS scores (letters)	Gain in postoperative ETDRS scores (letters)			Preoperative CRT (*μ*m)	Decrease in postoperative CRT (*μ*m)		
		One month	Three months	Six months		One month	Three months	Six months
27-gauge vitrectomy (100 eyes)	74.7 ± 12.5 (15-89)	4.7 ± 8.1	6.8 ± 9.4	7.8 ± 9.7	381.5 ± 84.0 (253–656)	−24.2 ± 50.0	−38.9 ± 64.3	−56.9 ± 48.1
25-gauge vitrectomy (100 eyes)	73.6 ± 13.3 (30-89)	1.1 ± 13.6	4.6 ± 13.4	6.4 ± 12.7	373.5 ± 97.3 (187–698)	−8.0 ± 48.6	−37.9 ± 91.4	−55.7 ± 93.1
*P* value	0.7298^a^	0.0421^a^	0.0835^a^	0.0569^a^	0.3850^a^	0.0354 ^a^	0.6059 ^a^	0.1725 ^a^

^a^Mann-Whitney test, *ETDRS* Early Treatment Diabetic Retinopathy Study, *CRT* central retinal thickness

## Discussion

We compared the postoperative outcomes of 27-G and 25-G vitrectomy performed for the treatment of idiopathic epiretinal membrane, with the following major findings. First, the time for performing 27-G vitrectomy was approximately 4 min longer than that for 25-G vitrectomy. Second, 1-month postoperative gain in ETDRS score was significantly better in the 27-G group than in the 25-G group (*P* = 0.0421). Three-month and 6-month postoperative ETDRS gain also tended to be better in the 27-G group, although the differences did not reach statistical significance (*P* = 0.0835, 0.0569). Third, low rates of hypotony (IOP ≤ 6 mmHg; 27-G vs. 25G: 2% vs. 6%) and ocular hypertension (4% vs. 11%) were observed on postoperative day 1 in both groups, and while the rates tended to be lower in the 27-G group, there were no significant differences. Mitsui et al. [6] performed vitrectomy on 74 eyes with epiretinal membrane using 25-G or 27-G instrument, and followed the eyes for 6 months. Their vitrectomy time was significantly longer with the 27-G instrument. The incidence of hypotony [IOP < 7 mmHg] on postoperative day 1 was high in both 25-G (35%) and 27-G groups (30%), with no significant difference. They observed no significant differences in postoperative visual acuity. Both our study and Mitsui’s study showed longer operative time when using the 27-G system. However, while we observed significant improvement in visual acuity on postoperative day 1, Mitsui et al. found no improvement. Also, the postoperative hypotony rate was much higher in Mitsui’s study than in our study. Thus, the novel findings of our study were early postoperative improvement of visual acuity and stabilized IOP when using the 27-G system.

Various factors that potentially affect postoperative visual acuity following vitrectomy for epiretinal membrane surgery will be discussed, including the gauge of surgical instrument, operation time, postoperative endophthalmitis, postoperative IOP, surgically induced astigmatism, postoperative central retinal thickness, and postoperative complications. Regarding the relationship between gauge of instrument and postoperative visual acuity, our study showed that mean ETDRS scores gained at 1 month after vitrectomy was significantly better in the 27-G group. In studies comparing 20-G with 25-G vitrectomy [12, 13], comparing 20-G or 23-G with 25-G vitrectomy [10], and comparing 23-G with 25-G vitrectomy [11], all reported earlier recovery of visual acuity when using 25-G vitrectomy. These results indicate that postoperative recovery of visual acuity is more rapid with smaller instrument gauge.

The operation time for vitrectomy in our series was 36.7 ± 12.8 min for 27-G and 32.7 ± 10.1 min for 25-G vitrectomy, with 27-G vitrectomy taking approximately 4 min longer (*P* = 0.0323). In the report of Mitsui et al. [6], the mean time of using the vitreous cutting was 9.9 ± 3.5 min with 27-G and 6.2 ± 2.7 min with 25-G instrument, and was also significantly longer when using the 27-G system (*P* < 0.0001). Sandali et al. [10] reported 30.15 ± 7.34 min for 23-G, and 31.53 ± 5.76 min for 25-G vitrectomy, with 25-G vitrectomy requiring approximately 2 min longer although there was no significant difference between the two. Rizzo et al. [13] reported 29.6 min for 20-G vitrectomy, which was 14 min longer than 15.6 min for 25-G vitrectomy (*P* < 0.01). These results indicate that more time is required for vitreous excision as the instrument gauge decreases, resulting in longer operation time.

One of the potential factors affecting recovery of visual acuity is postoperative inflammation. Inoue et al. [7] conducted an animal study to compare postoperative intraocular inflammation following 25-G, 23-G and 20-G vitrectomy, and reported that smaller gauge can minimize the inflammation associated with vitrectomy. In vitrectomy for epiretinal membrane, 25-G surgery is generally regarded to result in less postoperative inflammation than 20-G surgery [13], while no difference in anterior chamber flare between 25-G and 27-G surgery has also been reported [6]. Comparing 27-G, 25-G, 23-G and 20-G vitrectomy, although the available data suggest that postoperative inflammation decreases when using smaller gauge cutter, further studies are required to clarify this point.

The contribution of stabilized IOP after vitrectomy on rapid recovery of visual acuity has been studied in epiretinal membrane surgery. In the present series, all vitrectomies performed using 27-G and 25-G instruments were completed without suture. On postoperative day 1, the hypotony rates were 2% in the 27-G group and 6% in the 25-G group, while the ocular hypertension rates were 4% in the 27-G group and 11% in the 25-G group. Hypotony observed on postoperative day 1 was due to postoperative subclinical leakage. More stabilized IOP was obtained using 27-G instrument compared to 25-G instrument. In the study of Sandali et al. [10], the IOP on the first postoperative day increased significantly in the 20-G group (*P* < 0.001), but decreased significantly in the 23-G group (*P* = 0.073), while IOP did not change significantly in the 25-G group (*P* = 0.807). In the series reported by Kim et al. [11], intraoperative suturing of sclerotomy sites was required in 11.3% of the eyes in the 23-G group, whereas none of the eyes in the 25-G group needed suturing of sclerotomy (*P* < 0.002). Hypotony defined as IOP lower than 6 mmHg (1.9%) or intraocular pressure elevation over 30 mmHg (1.9%) was found on postoperative day 1 in the 23-G group, but not in the 25-G group. These findings indicate that use of smaller gauge achieves more stabilized IOP after vitrectomy, resulting in decreased frequencies of postoperative hypotony and ocular hypertension.

Surgically induced astigmatism and central retinal thickness that may affect recovery of visual acuity after vitrectomy have been compared among different systems. Regarding central retinal thickness, there was no difference between 25-G and 27-G vitrectomy [6]. In the present study, decrease in CRT was significantly greater in 27-G group compared to 25-G group at 1 month post-vitrectomy, but not significantly different at 3 and 6 months. Therefore, CRT decrease may have played a role in visual acuity improvement. Surgically induced astigmatism has been reported to be significantly less after 23-G and 25-G vitrectomy compared to 20-G vitrectomy [8, 9]. However, no difference in surgically induced astigmatism was observed between 25-G and 27-G vitrectomy in patients with epiretinal membrane [6]. These reports suggest that when 27-G, 25-G or 23-G vitrectomy is performed without suture, the risk of surgically induced astigmatism is low.

Finally, the impact of surgery-related complications on recovery of visual acuity after vitrectomy is discussed. In our study, the incidence of postoperative complications was low in both groups. Vitreous hemorrhage occurred in 1/100 eyes (1%) in the 27-G group, while retinal detachment and vitreous hemorrhage occurred in 1/100 eyes each (1%) in the 25-G group, with no significant difference. Sandali et al. [10] reported incidence of operative retinal breaks of 8.4% for 20-G, 6.1% for 23-G, and 2.2% for 25-G vitrectomy, showing a tendency of decrease in incidence with smaller gauge instrument but no significant difference. Hass et al. [26] performed 20-G and 23-G vitrectomy for epiretinal membrane, and reported incidence of 1.8% retinal detachments and 1.2% vitreous hemorrhages for 20-G, and 1.6% retinal detachment and 0% vitreous hemorrhage for 23-G vitrectomy, with no significant difference. These findings indicate that when comparing 27-G, 25-G and 23-G vitrectomy, smaller gauge is associated with lower risks of retinal breaks, retinal detachment and vitreous hemorrhage.

This study has some limitations. First, both the 25-G and 27-G vitrectomies were performed by two surgeons. However, the proportion of surgeries performed by each surgeon did not differ between 25-G and 27-G vitrectomies. Therefore, there is probably little surgeon-related effect. Second, the present research was a retrospective study. Further prospective study with larger number of cases is required to verify the present findings.

Having reviewed the potential factors that may influence postoperative visual acuity after epiretinal membrane surgery, stabilized IOP after surgery appears to be contribute to early improvement of CRT and early recovery of visual acuity. With respect to stabilized IOP after surgery, 27-G vitrectomy is a superior modality. The present study confirms that although 27-vitrectomy requires operation time of 4 min longer compared to 25-G vitrectomy, a low incidence of postoperative hypotony and ocular hypertension, few postoperative complications, and early postoperative recovery of visual acuity can be expected from this modality. MIVS performed as day surgery is increasingly being used in the world. Vitrectomy using the 27-G system has the advantages of earlier visual improvement and stabilized ocular pressure. This modality as day surgery is expected to gain global popularity.

## Conclusions

Although 27-G vitrectomy requires operation time of 4 min longer compared to 25-G vitrectomy for ERM surgery, using the 27-G system results in earlier recovery of visual acuity, CRT improvement and stabilized ocular pressure.

## Abbreviations

cpm: Cuts per min
G: Gauge
IOP: Intraocular pressure
MIVS: Microincision vitrectomy surgery
